# Increase in congenital syphilis in the European Union and European Economic Area, 2015 to 2024 – time to scale-up public health responses

**DOI:** 10.2807/1560-7917.ES.2026.31.38.2600240

**Published:** 2026-09-24

**Authors:** Otilia Mårdh, Disa Hansson, Katja Siling, Lina Nerlander, Csaba Ködmön, on behalf of the EU/EEA STI Network

**Affiliations:** 1 European Centre for Disease Prevention and Control (ECDC), Stockholm, Sweden; 2 The members of the network are listed in the Acknowledgements section at the end of the article.

**Keywords:** congenital syphilis, antenatal screening, syphilis, pregnancy, EU/EEA surveillance, elimination

## Abstract

**BACKGROUND:**

Congenital syphilis (CS) is under routine surveillance in the European Union and European Economic Area (EU/EEA). In 2024, notifications reached their highest level in a decade, coinciding with rising syphilis among women.

**AIM:**

We describe CS cases in 2024, trends from 2015 to 2024, associations between CS rates and syphilis among women of reproductive age, and public health implications.

**METHODS:**

Confirmed CS cases and syphilis notifications in 15–44-year-old women reported by EU/EEA countries between 2015 and 2024 were extracted from EpiPulse. For CS cases in 2024, we described key demographics and outcome (alive/deceased). Country-specific CS and syphilis rates per 100,000 were calculated using Eurostat denominators. We assessed associations between CS and syphilis in women using quasi-Poisson regression adjusting for population size and year.

**RESULTS:**

In 2024, 28 EU/EEA countries reported 140 confirmed CS cases, Bulgaria, Hungary and Portugal accounting for 62% (87/140). Most cases were diagnosed neonatally (86/99; 87%), and 11 of 58 were deceased. From 2015 to 2024, CS rates increased from 1.3 to 5.2 per 100,000 live births (23 countries) and syphilis rates among women aged 15–44 years from 1.9 to 5.3 per 100,000 (22 countries). Regression analyses showed significant positive associations between syphilis in women and CS rates in Germany and Hungary, whereas CS increases in Romania and Slovakia remained after adjustment for syphilis among women.

**CONCLUSION:**

The CS increase highlights prevention gaps. Strengthened surveillance, national audits of antenatal screening pathways, and testing policies adapted to evolving epidemiology are needed.

**Key public health message**

**What did you want to address in this study and why?**

Congenital syphilis occurs when syphilis is passed from a pregnant woman to her baby. It can be severe or fatal for the baby but is usually preventable with timely testing and treatment of the mother. In 2024, European countries reported the highest number of cases in the past decade, alongside increasing syphilis cases among women. We describe recent cases in babies and 10-year trends to better understand where prevention was missed.

**What have we learnt from this study?**

The number of congenital syphilis cases differed widely across the European Union and European Economic Area. In 2024, most cases came from three countries, while half of the countries reported no cases. Most babies were diagnosed during their first month of life, and among those with a known outcome, one in five had died. In some countries, increases in congenital syphilis were linked to more syphilis cases among women aged 15–44 years.

**What are the implications of your findings for public health?**

The increase in congenital syphilis shows that some infections during pregnancy are not being prevented. Looking closer at each case, and at how pregnant women are tested in each country can help identify where prevention can improve. In countries where syphilis is increasing among women, a second test later in pregnancy may be needed. Better surveillance and antenatal testing policies adapted to evolving epidemiology are needed.

## Introduction

Untreated or inadequately treated syphilis during pregnancy can lead to severe adverse fetal outcomes, including stillbirth, neonatal death and congenital infection [1]. These outcomes are preventable through routine antenatal syphilis screening and timely treatment appropriate to the stage of infection [2-4]. Congenital syphilis (CS), defined as infection of the fetus or infant resulting from vertical (transplacental) transmission of *Treponema pallidum* (*T. pallidum*) during any time of pregnancy, is under routine public health surveillance in the European Union and European Economic Area (EU/EEA) using standardised case definitions [5].

In 2022, the World Health Organization (WHO) estimated 700,000 cases of CS globally [6]. The European Region accounts for a small proportion of this burden (0.3%) and the WHO Regional Office for Europe has adopted an ambitious regional elimination target of ≤ 1 CS case per 100,000 live births by 2030 [7]. While most EU/EEA countries report low CS rates, and several already meet the target, recent data show a concerning rise. The overall EU/EEA rate has increased more than threefold over the past decade, from 1.3 per 100,000 live births in 2015 to 5.2 per 100,000 in 2024 in countries reporting consistently for this period [8]. Alongside the rise in CS, recent increases have been observed in syphilis notifications among women of reproductive age, notably since 2022 [9]. Similar parallel increases in CS and syphilis among women have been observed globally, including in Japan, where CS cases doubled between 2021 and 2023 alongside rising syphilis diagnoses among pregnant women [10], in Australia, where CS resurgence has been linked to rising syphilis among women of reproductive age [11], and in the United States, where CS rates have increased for more than a decade driven by rising maternal syphilis and persistent barriers to prenatal care and testing [12,13].

This study describes confirmed CS cases reported in the EU/EEA in 2024, analyses trends from 2015 to 2024, examines the association between CS and syphilis notifications among women of reproductive age, and outlines implications for public health action in the context of the WHO European Region’s 2030 CS elimination target.

## Methods

The EU/EEA countries report CS cases that meet laboratory criteria for a confirmed case (Box) to the European Centre for Disease Prevention and Control (ECDC) in a case-based format every year. However, not all countries submit data annually, Bulgaria and the Netherlands report only the number of cases without details on case characteristics, and Hungary also reports intrauterine deaths which are not included in the EU/EEA level case definition. Two countries reported a small number of probable and possible cases in 2024 (Poland: one probable and two possible; Sweden: one probable); those cases were not included in this analysis.

Box **Case definitions of congenital syphilis for surveillance at EU/EEA level**

**Clinical criteria** Any child < 2 years of age with at least one of the following:

· Hepatosplenomegaly
· Mucocutaneous lesions
· Condyloma lata
· Persistent rhinitis
· Jaundice
· Pseudoparalysis (due to periostitis and osteochondritis)
· Central nervous system involvement
· Anaemia
· Nephrotic syndrome
· Malnutrition

**Laboratory criteria**

For confirmed cases at least one of the following:

· demonstration of *Treponema pallidum* by dark-field microscopy in the umbilical cord, placenta, nasal discharge or skin lesion material
· demonstration of *T. pallidum* by DFA-TP in the umbilical cord, placenta, nasal discharge or skin lesion material
· detection of *T. pallidum*-specific IgM (FTA-abs, EIA) **and** a reactive non-treponemal test (VDRL, RPR) in the child’s serum

For probable cases at least one of the following:

· reactive VDRL-CSF test result
· reactive non-treponemal **and** treponemal serological tests in the mother’s serum
· infant’s non-treponemal antibody titre is at least fourfold higher than the mother’s antibody titre

**Epidemiological criteria** Any infant with an epidemiological link through human-to-human (vertical) transmission

**Case classification**

A. Possible case Not applicable.

B. Probable case Any infant or child meeting the clinical criteria **and** at least one of the following:

· an epidemiological link
· meeting the laboratory criteria for a probable case

C. Confirmed case Any infant meeting the laboratory criteria for case confirmation.

CSF: cerebrospinal fluid; DFA-TP: direct fluorescent antibody test for *Treponema pallidum*; EIA: enzyme immunoassay; EEA: European Economic Area; EU: European Union; FTA-abs: fluorescent treponemal antibody absorption test; IgM: immunoglobulin M; RPR: rapid plasma reagin test; VDRL: Venereal Disease Research Laboratory test.Currently, only confirmed cases that meet the laboratory criteria are expected to be reported at the EU/EEA level.

For this analysis, data on confirmed CS cases reported for the period 2015 to 2024 were retrieved from the EpiPulse system [14], using the date of diagnosis or, when unavailable, the date used for statistics, which is a date assigned by the reporting country as the closest available proxy for the date of diagnosis. We calculated national CS rates for countries with comprehensive surveillance using Eurostat annual live birth data as the denominator for each year over the period [15]. We extracted information on age at diagnosis (in months or, if not available, in years), sex at birth, and outcome (alive or deceased). For cases reported as deceased, the timing of death relative to the date of diagnosis cannot be determined from the available data. Maternal migrant status was inferred when the mother’s country of birth differed from the reporting country of the CS case.

Data on confirmed syphilis cases among women aged 15–44 years were extracted for the same period, and national notification rates per 100,000 population were calculated using Eurostat population denominators. We described trends over the period 2015 to 2024 using data from countries with comprehensive surveillance and consistent reporting.

The association between national CS rates and national syphilis notification rates among women aged 15–44 was assessed using regression analysis. Countries were excluded if they did not report data for all years from 2015 to 2024, or if they reported zero confirmed CS cases for more than five years during this period. Since the outcome represents counts of CS cases, we fitted a generalised linear model using a quasi-Poisson distribution with a log link to account for overdispersion, including population size as an offset to consider that the population size varies and to translate the results to rates. Additional details regarding the model are provided in the Supplement. Analyses were performed using R (version 4.4.2).

## Results

### Congenital syphilis notifications in 2024

**Figure 1:**
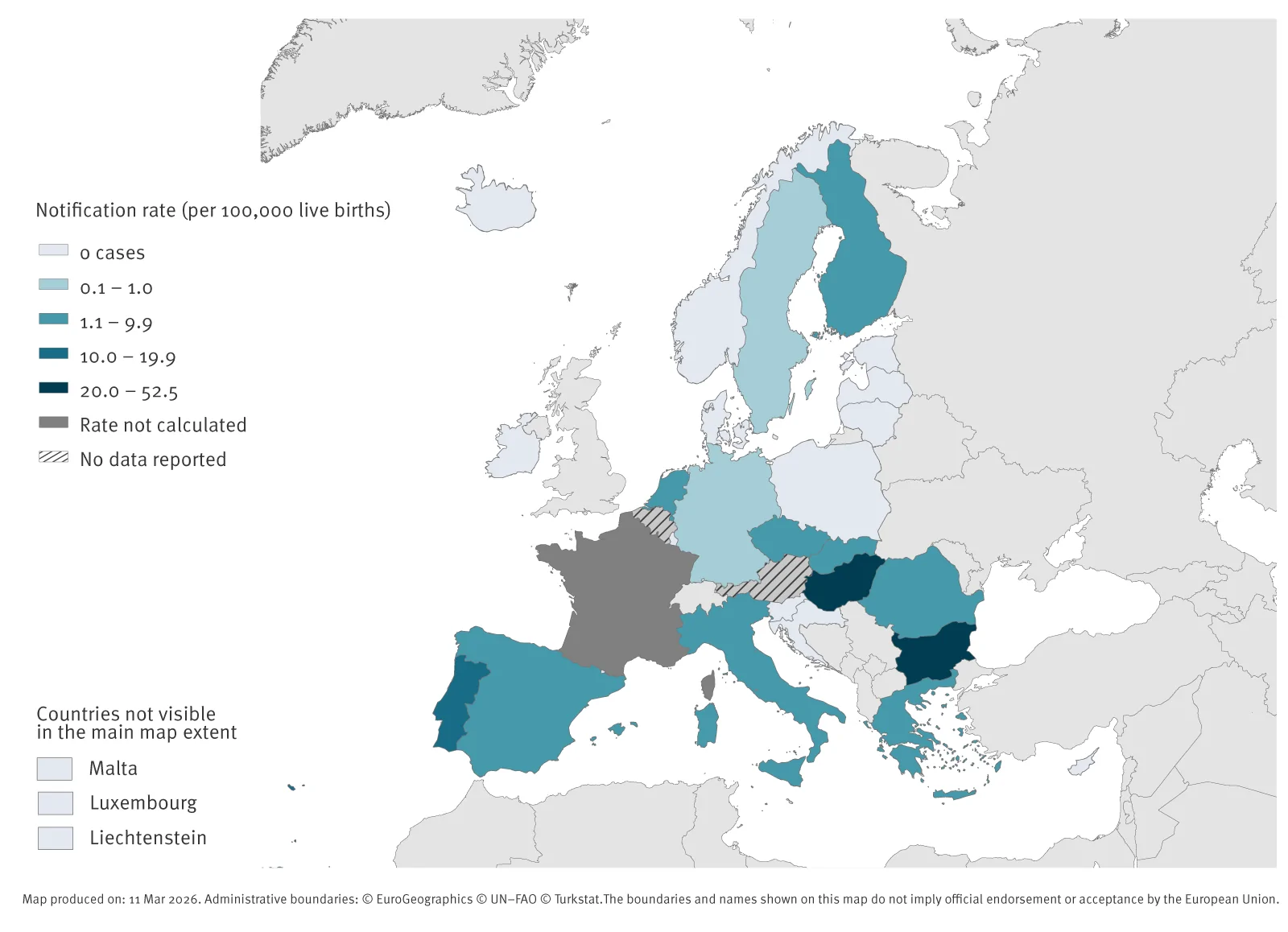
Confirmed congenital syphilis notification rates per 100,000 live births, EU/EEA, by country, 2024

EEA: European Economic Area; EU: European Union.

Data for 2024 from 28 EU/EEA countries (Austria and Belgium did not report) indicate 140 confirmed CS cases. Among countries with comprehensive surveillance that reported confirmed CS cases, national notification rates ranged from 0.9 to 52.5 per 100,000 live births (Table 1, Figure 1); a rate was not calculated for France, which implements sentinel surveillance. The rates were highest in Bulgaria and Hungary, at 52.5 and 47.9 per 100,000, respectively; together, these countries reported approximately half of all CS cases. Portugal also reported a high rate (17.5/100,000; 15 cases). Rates between 1.2 and 8.2 per 100,000 live births were reported by eight countries (41 cases in total). In contrast, two countries, Germany and Sweden, reported low notification rates of ≤ 1 per 100,000 live births (six cases in total). Fourteen countries reported zero confirmed cases in 2024.

**Table 1:** Confirmed congenital syphilis cases and notification rates, syphilis among women aged 15–44 years cases are notification rates, and antenatal screening policies, by date of diagnosis, EU/EEA, 2024 data (n = 140 congenital syphilis; n = 4,223 syphilis among women 15–44 years)

Country	Congenital syphilis		Age in months at diagnosis: range, median (total with data)	Sex: female/male (total with data)	Outcome: n deceased (total with data)	Mother's birth country different from reporting country: n (total with data)	Syphilis among women 15–44 years		Screening policy	Screening in 1st trimester	Repeat screening in 3rd trimester	Screening at delivery
	Cases	Rate/100,000					Cases	Rate/100,000				
Austria	NDR	NRC	NA				NDR	NRC	MAND	Y	NI	
Belgium	NDR	NRC	NA				285	NRC	UNIV	Y	RF	NI
Bulgaria	30	52.5	NDR				104	10.2	UNIV	Y	NI	YN
Croatia	0	0	NA				5	0.8	TARG	NI		
Cyprus	0	0	NA				5	2.5	UNIV	Y	RF	NI
Czechia	2	2.2	0–0, 0 (2)	1/1 (2)	NDR	1 (1)	82	4.3	MAND	Y	ALL	YN
Denmark	0	0	NA				76	6.9	UNIV	Y	NI	
Estonia	0	0	NA				7	2.8	UNIV	Y	NI	YN
Finland	1	2.3	NR	1/0 (1)	NDR		42	4.2	UNIV	Y	NI	
France	5	NRC	0–0, 0 (5)	3/2 (5)	2 (5)	0 (2)	308	NRC	UNIV	Y	NI	YN
Germany	6	0.9	NR	2/4 (6)	NDR		504	3.5	UNIV	Y	RF	NI
Greece	5	7	0–2, 0 (5)	4/1 (5)	0 (5)	NDR	40	2.3	UNIV	Y	ALL	YN
Hungary	42	47.9	0–16, 0 (42)	18/19 (37)	9 (9)	0 (21)	447	26.4	MAND	Y	NI	
Iceland	0	0	NA				10	12.9	UNIV	Y	NI	
Ireland	0	0	NA				61	5.6	UNIV	Y	RF	YNRF
Italy	7	1.8	0–8, 1 (7)	1/6 (7)	NDR		202	2.2	UNIV	Y	RF	NI
Latvia	0	0	NA				12	3.7	NR			
Liechtenstein	0	0	NA				0	0	UNIV	Y	NI	
Lithuania	0	0	NA				24	4.8	UNIV	Y	ALL	YN
Luxembourg	0	0	NA				19	13.7	UNIV	Y	RF	NI
Malta	0	0	NA				31	27.3	UNIV	Y	NI	YN
The Netherlands	2	1.2	NDR				76	NRC	UNIV	Y	NI	YN
Norway	0	0	NA				13	1.2	UNIV	Y	NI	
Poland	0	0	NA				Data not reported by sex and age		MAND	Y	RF	NI
Portugal	15	17.5	0–4, 0 (15)	5/10 (15)	0 (15)	8 (15)	415	23	UNIV	Y	ALL	YN
Romania	10	6.2	0–0, 0 (10)	3/7 (10)	0 (10)	0 (10)	146	4.4	UNIV	Y	ALL	NI
Slovakia	4	8.2	0–0, 0 (4)	2/2 (4)	0 (4)	NDR	133	13.3	UNIV	Y	ALL	NI
Slovenia	0	0	NA				11	3.2	UNIV	Y	NI	
Spain	10	3.1	0–2, 0 (9)	5/5 (10)	NDR	8 (10)	1,138	13.2	UNIV	Y	RF	YN
Sweden	1	1	0–0, 0 (1)	1/0 (1)	NDR		27	1.4	UNIV	Y	None^a^	NI
**EU/EEA**	**140**	**5.2^b^**	**0–16, 0 (99)^c^**	**NA**	**11 (58)**	**17 (63)**	**4,223**	**6.6^d^**	**NA **			

ALL: all pregnant women; EEA: European Economic Area; EU: European Union; MAND: mandatory screening (all pregnant women screened as part of antenatal care, with legislation in place enforcing it); NA: not applicable (e.g. countries with zero cases or no data reported); NDR: no data reported; NI: policy not indicated; NR: not reported; NRC: no population-based rate calculated (for France for congenital syphilis and for Belgium, France and the Netherlands for syphilis as surveillance coverage is not national); RF: restricted to women with identified risk factors; TARG: targeted screening (recommended for people with medical or epidemiological indications, recommended by a doctor, most often a gynaecologist, on a case-by-case basis); UNIV: universal screening (systematic voluntary testing offered to all pregnant women as part of routine care irrespective of individual risk factors. Consent may or may not be discussed); Y: yes; YN: yes if not tested before; YNRF: yes if not tested before and if identified risk factors.

^a^ There is no national policy but many of the regions in Sweden implement risk factor-based screening.

^b^ Rate calculated for 27 countries with comprehensive surveillance. The five cases from France are not included, as France implements a sentinel surveillance system for CS.

^c^ For seven more cases, age was reported in years: 1 year for the case in Finland and 0 years for the six cases in Germany.

^d^ Rate calculated for 24 countries that implement comprehensive surveillance and for which population denominator was known. They reported a total of 3,554 cases for 2024.

All countries applied the EU case definition for confirmed congenital syphilis, except Ireland, Hungary, Romania and the Netherlands, which used national definitions (e.g. inclusion of intrauterine deaths in Hungary). In addition to the 140 confirmed cases, Poland reported one probable and two possible cases, and Sweden reported one probable case.

Data visualisation was generated using the European Centre for Disease Prevention and Control Map Maker tool [32]. Rates are shown only for countries with comprehensive surveillance that reported data for 2024.

### Characteristics of reported confirmed congenital syphilis cases, 2024

Age at diagnosis was available for 106 of 140 confirmed CS cases (Table 1). Among the 99 cases with age reported in months, 86 (86.9%) were diagnosed in the neonatal period (0 months), 12 between 1 and 8 months, and one case at 16 months. For seven cases, age was reported in years: 0 years for six cases and 1 year for one case. Sex at birth was available for 103 cases, of whom 46 (44.7%) were female. Outcome was known for 58 cases; 11 were reported as deceased, all diagnosed within the first month of life. Maternal region of birth was reported for 63 cases; among these, 17 mothers were born outside the reporting country, in South America (13/17), another EU/EEA country (2/17), a non-EU/EEA European country (1/17) and the Caribbean (1/17). For context, national antenatal syphilis screening policies in the reporting countries are presented in Table 1.

### Trends in congenital syphilis notifications and syphilis among women, 2015–2024

For trend analyses (Table 2), we included only the 23 countries with comprehensive CS surveillance and consistent reporting for all years 2015 to 2024. During this period, 580 confirmed CS cases were reported. The EU/EEA CS rate increased from 1.3 per 100,000 live births in 2015 (35 cases; eight countries) to 5.2 per 100,000 in 2024 (122 cases; 10 countries). Following a gradual increase between 2015 and 2019 and a decline in 2020 and 2021, CS notifications rose from 2022 onwards, and the EU/EEA rate almost doubled between 2022 and 2024. In 2024, seven countries reported higher case counts than in 2022 (Bulgaria, Czechia, Germany, Hungary, the Netherlands, Romania and Spain). The largest increase was observed in Hungary. In addition, although not included in Table 2, higher case counts were also reported in France (five cases in 2024 vs three in 2022; data derived from sentinel surveillance and therefore not nationally representative) and Italy (seven cases in 2024 vs two in 2022; data available for 2018 to 2024).

**Table 2:** Confirmed congenital syphilis cases and notification rates per 100,000 live births by date of diagnosis, EU/EEA^a^, 2015–2024 (n = 580)

Country	2015		2016		2017		2018		2019		2020		2021		2022		2023		2024	
	Cases	Rate	Cases	Rate	Cases	Rate	Cases	Rate	Cases	Rate	Cases	Rate	Cases	Rate	Cases	Rate	Cases	Rate	Cases	Rate
Bulgaria	10	15.2	13	20	14	21.9	25	40.2	37	60.1	16	27.1	13	22.2	24	42.4	13	22.7	30	52.5
Croatia	0	0.0	0	0.0	0	0.0	0	0.0	0	0.0	0	0.0	0	0.0	0	0.0	1	3.1	0	0.0
Cyprus	0	0.0	0	0.0	0	0.0	0	0.0	0	0.0	0	0.0	0	0.0	2	19.6	0	0.0	0	0.0
Czechia	4	3.6	1	0.9	1	0.9	0	0.0	3	2.7	4	3.6	1	0.9	0	0.0	0	0.0	2	2.2
Denmark	0	0.0	1	1.6	0	0.0	0	0.0	1	1.6	0	0.0	0	0.0	1	1.7	0	0.0	0	0.0
Estonia	0	0.0	0	0.0	0	0.0	0	0.0	0	0.0	0	0.0	0	0.0	0	0.0	0	0.0	0	0.0
Germany	3	0.4	2	0.3	3	0.4	3	0.4	3	0.4	6	0.8	1	0.1	2	0.3	3	0.4	6	0.9
Hungary	0	0.0	2	2.1	3	3.2	5	5.3	3	3.2	3	3.2	12	12.8	8	8.9	13	14.8	42	47.9
Iceland	0	0.0	0	0.0	0	0.0	0	0.0	0	0.0	0	0.0	0	0.0	0	0.0	0	0.0	0	0.0
Ireland	0	0.0	0	0.0	1	1.6	0	0.0	1	1.7	0	0.0	0	0.0	1	1.8	0	0.0	0	0.0
Latvia	0	0.0	0	0.0	1	4.8	1	5.2	0	0.0	0	0.0	0	0.0	0	0.0	0	0.0	0	0.0
Lithuania	3	9.5	0	0.0	1	3.5	0	0.0	0	0.0	0	0.0	0	0.0	0	0.0	1	4.8	0	0.0
Luxembourg	0	0.0	0	0.0	0	0.0	0	0.0	0	0.0	0	0.0	0	0.0	0	0.0	0	0.0	0	0.0
Malta	0	0.0	0	0.0	0	0.0	0	0.0	0	0.0	0	0.0	0	0.0	0	0.0	0	0.0	0	0.0
The Netherlands	0	0.0	1	0.6	3	1.8	1	0.6	1	0.6	0	0.0	1	0.6	0	0.0	0	0.0	2	1.2
Norway	0	0.0	0	0.0	0	0.0	0	0.0	0	0.0	0	0.0	0	0.0	0	0.0	0	0.0	0	0.0
Poland	4	1.1	6	1.6	1	0.2	2	0.5	3	0.8	0	0.0	0	0.0	1	0.3	1	0.4	0	0.0
Portugal	5	5.8	2	2.3	4	4.6	4	4.6	13	15	7	8.3	15	18.8	16	19.1	15	17.5	15	17.5
Romania	5	2.5	4	1.9	6	2.8	4	2.0	0	0.0	2	1.0	1	0.5	3	1.7	9	5.9	10	6.2
Slovakia	0	0.0	0	0.0	0	0.0	2	3.5	1	1.8	1	1.8	0	0.0	6	11.4	4	8.2	4	8.2
Slovenia	0	0.0	0	0.0	0	0.0	0	0.0	0	0.0	0	0.0	0	0.0	0	0.0	0	0.0	0	0.0
Spain	1	0.2	4	1.0	2	0.5	5	1.3	1	0.3	0	0.0	5	1.5	2	0.6	7	2.2	10	3.1
Sweden	0	0.0	2	1.7	0	0.0	2	1.7	1	0.9	0	0.0	0	0.0	2	1.9	0	0.0	1	1.0
**EU/EEA (23 countries)**	**35**	**1.3**	**38**	**1.3**	**40**	**1.4**	**54**	**2.0**	**68**	**2.5**	**39**	**1.5**	**49**	**1.8**	**68**	**2.7**	**67**	**2.8**	**122**	**5.2**

^a^ Countries with comprehensive surveillance and consistent reporting.

Over the same period, data on syphilis notifications from 22 of the 30 EU/EEA countries with comprehensive surveillance and consistent reporting (i.e. excluding Austria, Belgium, Bulgaria, France, Liechtenstein, the Netherlands, Poland and Spain) show that rates among women aged 15–44 years increased, with a steeper rise from 2022 onwards (data not shown). In 2024, the EU/EEA notification rate, calculated from data reported by these 22 countries, reached 5.3 per 100,000 women aged 15–44 years, representing a 183% increase compared with 2015. Most women diagnosed with syphilis were aged 20–34 years, the peak reproductive age.

**Figure 2:**
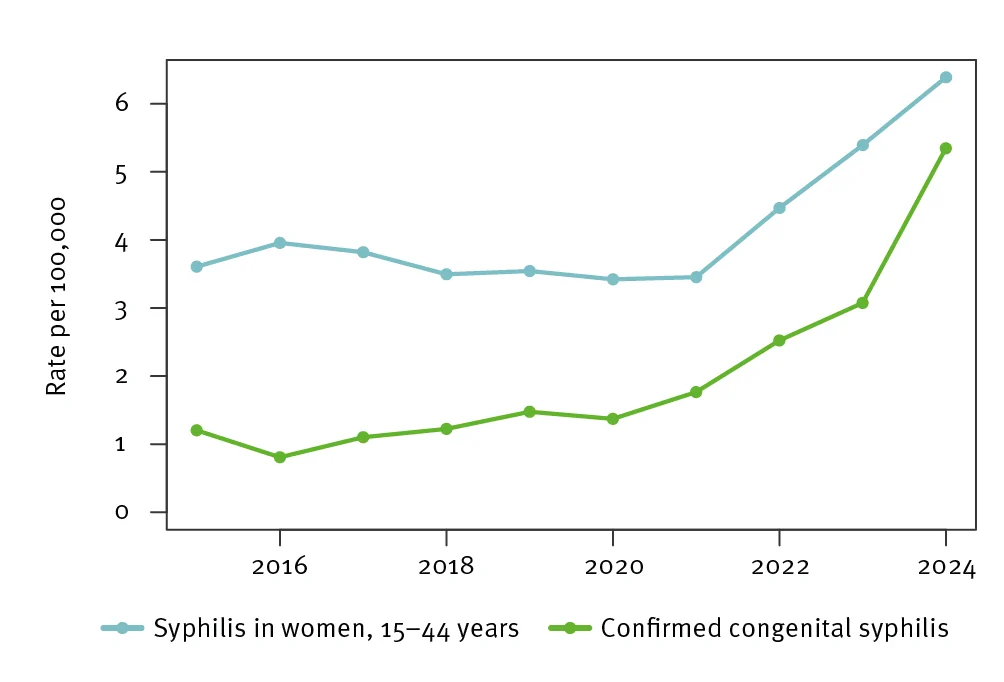
Trends in syphilis notification rates among women aged 15–44 years per 100,000 population and confirmed congenital syphilis notification rates per 100,000 live births, EU/EEA^a^, 2015–2024 (n = 12,937 syphilis among women 15–44 years; n = 318 congenital syphilis)

^a^ Countries with comprehensive surveillance and consistent reporting of both conditions: Croatia, Cyprus, Czechia, Denmark, Estonia, Germany, Hungary, Iceland, Latvia, Lithuania, Luxembourg, Malta, Norway, Portugal, Romania, Slovakia, Slovenia and Sweden.

Figure 2 presents trends in syphilis notifications among women aged 15–44 years and confirmed CS rates in the 18 EU/EEA countries with comprehensive surveillance and consistent reporting of both conditions throughout the period 2015 to 2024. In these countries, syphilis rates among women were relatively stable until 2021 and increased thereafter. The CS rates followed a similar pattern, with increases observed from 2021 onwards and a marked rise between 2022 and 2024.

### Association between syphilis among women and congenital syphilis rates

**Figure 3:**
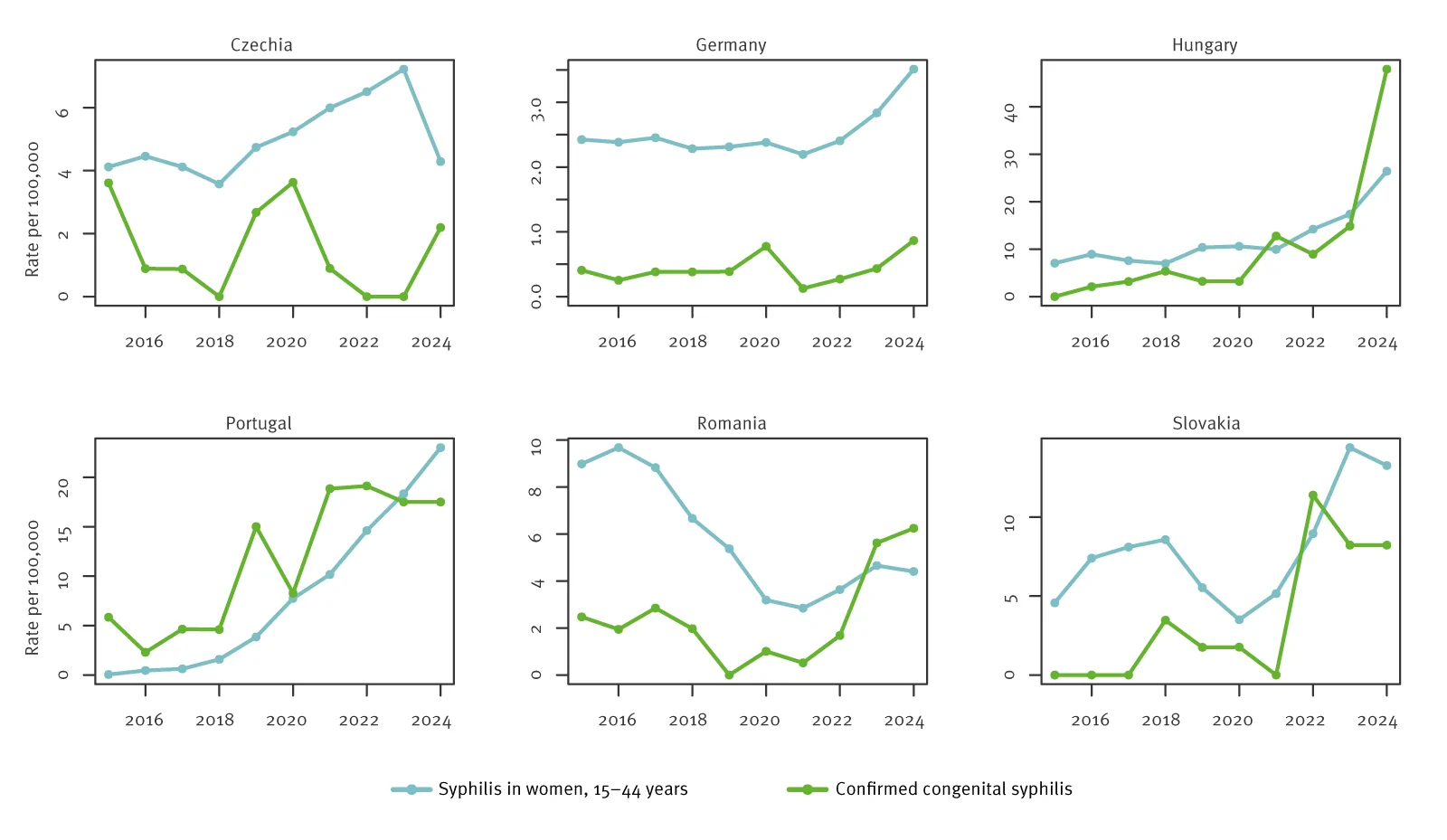
Trends in syphilis notification rates among women aged 15–44 years per 100,000 population and confirmed congenital syphilis notification rates and per 100,000 live births, in six countries, 2015–2024 (n = 11,134 syphilis among women 15–44 years; n = 297 congenital syphilis)

Six countries, Czechia, Germany, Hungary, Portugal, Romania and Slovakia, consistently reported CS and syphilis data between 2015 and 2024 and had CS case counts above zero in the majority of reporting years. Trends in syphilis among women aged 15–44 and CS notifications for these countries are shown in Figure 3.

Results from the quasi-Poisson regression models evaluating the association between CS rates and syphilis notification rates among women aged 15–44 years are presented in Table 3.

**Table 3:** Association between syphilis notification rates among women aged 15–44 years per 100,000 population and confirmed congenital syphilis notification rates per 100,000 live births, 2015–2024 (n = 11,134 syphilis among women 15–44 years; n = 297 congenital syphilis)

Country	Change in CS rate per one-unit increase in syphilis rate among women 15–44 years			Annual multiplicative change in CS rates (IRR per year), adjusted for syphilis rates among women aged 15–44 years		
	IRR	95% CI	p value	IRR	95% CI	p value
Czechia	0.66	0.15–2.87	0.501	0.96	0.63–1.47	0.809
Germany	2.48	**1.36–4.52**	**0.012**	0.98	0.90–1.06	0.534
Hungary	1.10	**1.01–1.21**	**0.039**	1.18	0.90–1.54	0.180
Portugal	0.93	0.77–1.13	0.406	1.46	0.81–2.64	0.162
Romania	1.70	0.98–2.95	0.056	1.56	**1.09–2.25**	**0.025**
Slovakia	0.88	0.61–1.26	0.388	1.63	**1.01–2.64**	**0.048**

CI: confidence interval; CS: congenital syphilis; IRR: incidence rate ratio.

Associations were estimated using quasi-Poisson regression models with a log link. An IRR > 1 indicates an increase in CS rates; for example, an IRR of 1.70 corresponds to a 70% annual increase. Statistically significant associations (p < 0.05) are highlighted in bold.

Across countries, the estimated effect of syphilis notification rates among women aged 15–44 years on CS rates varied substantially. In Germany and Hungary, higher syphilis rates were significantly associated with higher CS rates. In Germany, each unit increase in the syphilis rate corresponded to a more than twofold increase in the CS rate (incidence rate ratio (IRR) = 2.48; 95% confidence interval (CI): 1.36–4.52; p = 0.012). Because the model detects proportional increases at any incidence level, the association is evident despite Germany’s low baseline levels. Hungary showed a smaller but still statistically significant association (IRR = 1.10; 95% CI: 1.01–1.21; p = 0.039). Romania also showed a positive association (IRR = 1.70; 95% CI: 0.98–2.95; p = 0.056), although this did not reach statistical significance. No significant association was observed in Czechia, Portugal or Slovakia.

The CS trends over time also varied by country. Romania had a statistically significant increase in CS rate over time (IRR = 1.56; 95% CI: 1.09–2.25; p = 0.025) after adjustment for syphilis rate among women aged 15–44 years. Slovakia also had a significant upward trend (IRR = 1.63; 95% CI: 1.01–2.64; p = 0.048), while Portugal had an elevated but non-significant time effect (IRR = 1.46; 95% CI: 0.81–2.64; p = 0.162). For Germany, Czechia and Hungary, no statistically significant linear time trend was detected after adjusting for syphilis rates among women aged 15–44 years, indicating no detectable linear change in CS rates during the study period. Even though an increase in CS cases was visible in Hungary, this pattern was not captured as a significant linear time effect once syphilis rates were included in the model, reflecting the difficulty of detecting a consistent temporal effect when recent increases are sharp and nonlinear.

## Discussion

The 2024 data show the highest number of confirmed CS cases reported in the EU/EEA in the past decade, with an overall notification rate approximately four times higher than in 2015. Although CS is preventable through timely antenatal screening and appropriate treatment, this upsurge signals gaps along the prevention pathway.

Bulgaria, Hungary and Portugal, countries that together account for ca 6% of all live births in the EU/EEA and have consistently reported among the highest CS rates, contributed a substantial proportion of reported cases in 2024 (ca 62%; data not shown). However, increases in CS were also observed in countries with previously low incidence. This suggests that the recent rise is not confined to countries with a historically higher incidence and may reflect broader system-level challenges affecting antenatal screening quality, timeliness or treatment pathways across the EU/EEA. As epidemiological contexts evolve, screening strategies may require reassessment to ensure their quality and effectiveness remain fit for purpose.

The recent increase in CS coincided with a rise in syphilis notifications among women across the EU/EEA since 2022 and requires continued close monitoring. While universal first-trimester syphilis screening is recommended throughout the EU/EEA, and about half of the countries also recommend repeat screening later in pregnancy, data on implementation and coverage are limited [16]. In 2024, only five countries reported data on antenatal syphilis screening coverage, ranging from > 95% in Denmark, France, Germany and the Netherlands to ca 50% in Portugal. Notably, 59 CS cases occurred in countries with either universal or risk-based third-trimester screening policies in place. Given that identifying re-infection risk factors in pregnant women or their partners has proven challenging [17], universal repeat screening in the third trimester may be a safer approach in settings with rising syphilis notifications. Ensuring that sexual partners of pregnant women diagnosed with syphilis are tested and treated is also essential to prevent maternal re-infections and community transmission. Systematic national audits of antenatal screening and treatment pathways, including case-by-case reviews of all vertical transmission events, are essential to identify missed prevention opportunities and to inform adjustments to policies and guidelines [18,19].

Most CS cases in 2024 were diagnosed in the neonatal period, although some were diagnosed later in infancy, underscoring the importance of effective postnatal follow-up of exposed infants [20]. Outcome information was limited, but all 11 reported deaths had an age recorded as zero months, suggesting the possibility that some may have been stillbirths, although the EU case definition does not include stillbirths. Indeed, among nine fatal cases in Hungary, seven were intrauterine deaths and two postnatal deaths. Overall, these findings highlight the severe consequences of untreated maternal syphilis and the need for timely detection and treatment [21].

Available data from notified cases suggest that CS prevention disproportionately fails among pregnant people born outside the reporting country, probably reflecting barriers to early antenatal care and timely treatment among migrant communities [18]. Additional sources indicate that pregnancies among people who are homeless, experience drug dependence, are minors, or living in difficult socioeconomic conditions may be at increased risk of CS [8]. Improving outreach, providing social service support and culturally appropriate healthcare, and removing legal or administrative obstacles to early antenatal care are essential to preventing CS in these populations [2].

The 140 confirmed CS cases reported for 2024 probably underestimate the true incidence of syphilis-related adverse pregnancy outcomes in the EU/EEA. The current EU case definition excludes stillbirths and pregnancy losses (estimated at 21% of untreated syphilis in pregnancy [22]) and infections diagnosed in children older than 2 years. It also requires laboratory confirmation based on tests in the child that may not be routinely performed in clinical practice [23]. A 2025 ECDC survey identified additional diagnostic and reporting gaps, including difficulties in linking laboratory and clinical data, inconsistent data collection, and reporting limitations related to data protection regulations [24]. While Ireland has updated its case definition to align with current diagnostic practice [25], the ECDC is planning EU-level revisions to improve sensitivity and cross-country comparability.

The observed CS patterns align with broader increases in syphilis among heterosexual populations in the EU/EEA [9], potentially expanding the number of women at risk during pregnancy. Country-level regression analyses indicate heterogeneous associations between CS rates and syphilis rates among women aged 15–44 years. Positive associations were observed in several countries, including Germany and Hungary, although very low CS incidence in some settings (such as in Germany) warrants cautious interpretation, as small baseline numbers can exaggerate random fluctuations. In countries with higher CS incidence, a positive association is more likely to signal missed prevention opportunities in antenatal care.

Temporal analyses indicated increasing CS trends in Romania and Slovakia, while trends in Portugal were elevated but non-significant. Such patterns may reflect a range of potential factors, including variations in antenatal care performance, testing coverage, treatment timeliness, or changes in reporting practices. Although the specific drivers remain unclear, these upward trends are concerning, as even modest increases may signal emerging vulnerabilities in prevention efforts. The absence of association in other countries could reflect differences in screening coverage, reporting practices, or more effective antenatal screening and treatment interventions despite high rates of syphilis. However, where absolute CS rates are very low, the absence of an association may simply reflect the small number rather than true differences in programme performance.

Interpreting these results requires caution. A robust correlation analysis depends on well-performing surveillance systems for both CS and syphilis, with high completeness, consistent reporting, and a sensitive case definition for CS. Variation in any of these elements may weaken observed associations and could partly explain the heterogeneous patterns across countries.

The extent to which the COVID-19 pandemic influenced CS and syphilis trends remains unclear. The EU/EEA data showed a decline in both CS and syphilis notifications among women in 2020 and 2021, following a peak in 2019. The COVID-19 pandemic has been recognised as affecting STI surveillance quality through under-reporting and under-ascertainment, related to disruptions to testing, diagnosis and reporting capacities [26,27]. Reductions in STI testing activity during 2020 were reported by service providers in the WHO European Region; however, data on antenatal syphilis screening were not available [27]. An ECDC enquiry of the STI Network (2022 survey, data not shown) indicated that syphilis surveillance was affected by the COVID-19 pandemic in 14 of 22 countries reporting data for 2020, whereas 15 of 16 countries reported that there was no impact on CS data. Therefore, an accurate assessment of the impact of the pandemic, including potential effects on antenatal screening and CS case detection and reporting, is not supported by the available data, and is acknowledged as a limitation.

Despite the recent increases, our analysis shows that CS elimination is achievable in the EU/EEA: 16 countries in 2024 reported either zero cases or notification rates at or below the 2030 WHO European Region elimination threshold. Several countries with higher CS burden have responded by adapting screening policies to changing epidemiological context. In Hungary, additional measures were introduced in 2025, including universal rapid testing for all women in labour, followed by confirmatory laboratory investigation for mothers and infants when necessary [28,29]. Healthcare professionals were alerted about the rising number of CS cases and informed about measures to improve prenatal care (e.g. repeat testing, follow-up of seropositive pregnant women, and outreach to high-risk persons or groups). Portugal has also strengthened prevention efforts. Since 2015, the Programa Nacional de Vigilância da Gravidez de Baixo Risco (National Low-Risk Pregnancy Surveillance Programme) has reinforced early antenatal booking and ensured systematic first-trimester screening. Updated national recommendations introduced repeat serological testing in the third trimester and at delivery for women with identified risk factors [30], aiming to reduce missed diagnoses and ensure timely treatment. During the period under analysis, national surveillance was also strengthened to improve integration of laboratory results into routine CS epidemiological surveillance. In Croatia, despite a lower CS burden, a transition from targeted screening to universal antenatal screening is being considered within the professional community [23]. In September 2026, the ECDC and the European AIDS Clinical Society have jointly published European standards for antenatal care, aiming to help reverse current trends and support progress towards CS elimination by 2030 [31].

## Conclusions

Overall, these findings highlight the need for renewed public health action and for screening policies that reflect current epidemiological trends. Equitable access to high-quality antenatal care and effective implementation of screening policies, as well as a regular audit of syphilis screening coverage among pregnant women is also critical. Robust surveillance and improved diagnostic capacity are essential to better characterise the true burden of CS and identify gaps in prevention. 

## Data Availability

Data can be accessed on request through: Access to EU/EEA surveillance data for third parties.

## Supplementary Data

162000 Supplement
