# Supplementary material for: Increase in congenital syphilis in the European Union and European Economic Area, 2015 to 2024 – time to scale-up public health responses
Source: Euro Surveill. 2026 Sep 24;31(38):2600240. doi: 10.2807/1560-7917.ES.2026.31.38.2600240 (PMC13615387; doi:10.2807/1560-7917.ES.2026.31.38.2600240)
Supplement: Supplementary file 1 [file 26-00240_MARDH_Supplement.pdf]

This supplementary material is hosted by *Eurosurveillance* as supporting information alongside the article *Upsurge in congenital syphilis in EU/EEA, 2015-2024 - time to scale-up public health responses,* on behalf of the authors, who remain responsible for the accuracy and appropriateness of the content. The same standards for ethics, copyright, attributions and permissions as for the article apply. Supplements are not edited by *Eurosurveillance* and the journal is not responsible for the maintenance of any links or email addresses provided therein

Supplementary material – methods

Correlation and regression analysis

To investigate the association between reported rates of congenital syphilis and syphilis during 2015–2024, under the assumption that syphilis rates influence congenital syphilis rates, we used a quasi-Poisson regression model.

Before conducting the analysis, the two series were inspected for completeness: countries with missing values the last 10 years and countries with majority zero cases (more than 5 years with zero CS cases) were removed. The outcome variable was the annual number of congenital syphilis cases per country. The predictor variables were the annual syphilis rate (notifications per 100 000 women 15–44 years), and a continuous time variable representing years since 2014. To model congenital syphilis rates per 100 000 live births, we included an offset term equal to the log of live births divided by 100 000, allowing interpretation of coefficients as incidence rate ratios (IRR).

For each country, the model was specified as:

$$\log(\mu_t) = \beta_0 + \beta_1 \times \text{Syphilis}_t + \beta_2 \times t + \log(\text{Population}_t).$$

where μ_t is the expected count of congenital syphilis cases in year *t*; Syphilis_t is the syphilis notifications per 100 000 women aged 15-44 years; *t* is years since 2014; and Population_t is the number of live births divided by 100 000 for year *t*. The log link ensures multiplicative effects on the rate scale. Incidence rate ratios (IRRs) were obtained by exponentiating model coefficients. Because quasi-Poisson models do not rely on a full likelihood, 95% confidence intervals were calculated using Wald confidence intervals based on the estimated standard errors and the t-distribution with residual degrees of freedom.

Sensitivity analysis: negative binomial regression

As a sensitivity analysis, negative binomial models were fitted to assess the degree of overdispersion. The estimated overdispersion θ was large for all countries but one, indicating that the negative binomial model approached a Poisson variance structure. These findings support the use of a Poisson modelling framework. Therefore, the quasi-Poisson model was used as the main analytical approach, as it allows for potential overdispersion while preserving the interpretation of effects on the rate scale.

The estimated overdispersion parameter from the negative binomial regression model is presented in the table below.

Table: Estimated overdispersion parameter theta for the negative binomial regression model.

Country	Theta
Czechia	3
Germany	643 589
Hungary	115 924
Portugal	112 581
Romania	90 291
Slovakia	27 318

Analyses were performed using R (version 4.4.2).
